# Signature Arsenic Detoxification Pathways in *Halomonas* sp. Strain GFAJ-1

**DOI:** 10.1128/mBio.00515-18

**Published:** 2018-05-01

**Authors:** Shuangju Wu, Lianrong Wang, Rui Gan, Tong Tong, Hao Bian, Zhiqiang Li, Shiming Du, Zixin Deng, Shi Chen

**Affiliations:** a Key Laboratory of Combinatorial Biosynthesis and Drug Discovery, Ministry of Education, School of Pharmaceutical Sciences, Zhongnan Hospital, Wuhan University, Wuhan, China; b Taihe Hospital, Hubei University of Medicine, Shiyan, Hubei, China; c State Key Laboratory of Microbial Metabolism, Joint International Laboratory on Metabolic & Developmental Sciences, School of Life Sciences & Biotechnology, Shanghai Jiao Tong University, Shanghai, China; Oregon State University

**Keywords:** GFAJ-1, arsenate efflux, arsenic resistance, genome mapping

## Abstract

Since the original report that *Halomonas* sp. strain GFAJ-1 was capable of using arsenic instead of phosphorus to sustain growth, additional studies have been conducted, and GFAJ-1 is now considered a highly arsenic-resistant but phosphorus-dependent bacterium. However, the mechanisms supporting the extreme arsenic resistance of the GFAJ-1 strain remain unknown. In this study, we show that GFAJ-1 has multiple distinct arsenic resistance mechanisms. It lacks the genes to reduce arsenate, which is the essential step in the well-characterized resistance mechanism of arsenate reduction coupled to arsenite extrusion. Instead, GFAJ-1 has two arsenic resistance operons, *arsH1*-*acr3*-*2*-*arsH2* and *mfs1*-*mfs2*-*gapdh*, enabling tolerance to high levels of arsenate. *mfs2* and *gapdh* encode proteins homologous to Pseudomonas aeruginosa ArsJ and glyceraldehyde-3-phosphate dehydrogenase (GAPDH), respectively, which constitute the equivalent of an As(V) efflux system to catalyze the transformation of inorganic arsenate to pentavalent organoarsenical 1-arseno-3-phosphoglycerate and its subsequent extrusion. Surprisingly, the *arsH1*-*acr3*-*2*-*arsH2* operon seems to consist of typical arsenite resistance genes, but this operon is sufficient to confer both arsenite and arsenate resistance on Escherichia coli AW3110 even in the absence of arsenate reductase, suggesting a novel pathway of arsenic detoxification. The simultaneous occurrence of these two unusual detoxification mechanisms enables the adaptation of strain GFAJ-1 to the particularly arsenic-rich environment of Mono Lake.

## IMPORTANCE

*Halomonas* sp. strain GFAJ-1 was previously reported to use arsenic as a substitute for phosphorus to sustain life under phosphate-limited conditions. Although this claim was later undermined by several groups, how GFAJ-1 can thrive in environments with high arsenic concentrations remains unclear. Here, we determined that this ability can be attributed to the possession of two arsenic detoxification operons, *arsH1*-*acr3*-*2*-*arsH2* and *mfs1*-*mfs2*-*gapdh*. *mfs2* and *gapdh* encode proteins homologous to ArsJ and GAPDH in Pseudomonas aeruginosa; these proteins create an arsenate efflux pathway to reduce cellular arsenate accumulation. Interestingly, the combination of *acr3*-*2* with either *arsH* gene was sufficient to confer resistance to both arsenite and arsenate in E. coli AW3110, even in the absence of arsenate reductase, suggesting a new strategy for bacterial arsenic detoxification. This study concludes that the survival of GFAJ-1 in high arsenic concentrations is attributable to the cooccurrence of these two unusual arsenic detoxification mechanisms.

## INTRODUCTION

Arsenic is a highly toxic metalloid that poses a serious threat to our drinking water and food supply. Environmental arsenic occurs mainly as inorganic forms, such as trivalent arsenite [As(III)] and pentavalent arsenate [As(V)]. As(III) is reported to be on average 100 times more toxic than As(V) for most biological systems, in large part because the trivalent species reacts with active sulfhydryl groups in many enzymes, thereby impairing their function (1). The lower toxicity of arsenate results primarily from its physiochemical similarities to inorganic phosphate, although these similarities also lead to the indiscriminate acquisition of toxic arsenic into cells by cellular phosphate uptake systems. Once inside the cell, arsenate can compete with phosphate in phosphorylation reactions, but the products are less stable and dissociate rapidly (2, 3).

To survive in arsenic-rich environments, organisms have developed multiple metabolic strategies for detoxification, including the arsenic resistance (*ars*) system, which reduces cytoplasmic toxic As(V) to As(III); the arsenite oxidation (*aio*) system, which oxidizes As(III) to As(V); the arsenate respiration (*arr*) system, which is associated with respiration and reduces As(V) to As(III); and the arsenic methylation (*arsM*) system, which converts inorganic arsenic into volatile derivatives (4–8). *ars* is the most extensively studied arsenic detoxification system; the mechanism consists of (i) the uptake of arsenate by phosphate transporters and uptake of arsenite by aquaglyceroporins, (ii) transformation of As(V) to As(III) by arsenate reductases, and (iii) extrusion of As(III) by arsenite efflux permeases (9). The minimal *ars* constituents usually include either three (*arsRBC*) or five (*arsRDABC*) genes, which are often organized in operons (10). ArsR is an arsenite-responsive repressor that controls the level of *ars* operon transcription. The activity of ArsB, a transmembrane As(III) efflux permease that exports arsenite from the cytosol, can be ATP independent or require the help of ArsA, an arsenite-stimulated ATPase tightly bound to ArsB (11). ArsD was first characterized as a weak As(III)-responsive transcriptional regulator, but recent work has reported that it functions as a metallochaperone, transferring As(III) to the ArsAB efflux pump and thus enhancing arsenic extrusion (12, 13). ArsC is an arsenate reductase that catalyzes the conversion of As(V) to As(III), which can then be expelled from the cells. In addition to the above-mentioned two typical arsenic resistance operons, a broad diversity of *ars* operons has been characterized in different species (14–18).

*Halomonas* sp. strain GFAJ-1 (ATCC BAA-2256), which was isolated from the arsenic-rich sediments of Mono Lake in eastern California, was claimed to be able to use arsenic instead of phosphorus in biomolecules, specifically DNA, to sustain its growth (19). Although arsenate and phosphate share striking similarities, e.g., nearly identical pK_a_ values, similarly charged oxygen atoms, and comparable thermochemical radii, this conclusion was greeted with considerable skepticism because of the following. (i) Inorganic As(V) can be easily reduced to trivalent As(III) in physiological conditions. (ii) Arsenate esters are extremely unstable in aqueous solution and hydrolyze spontaneously. This “replacement of phosphorus by arsenic” claim challenged the physiological role of phosphorus and therefore triggered multiple investigations to prove the presence or absence of arsenate in DNA. A later growth analysis showed that the GFAJ-1 strain can grow at low phosphate concentrations (1.7 μM) but not in phosphorus-depleted (<0.3 μM) medium, even when supplemented with sufficient arsenate, confirming that arsenate does not contribute to the growth of GFAJ-1 (20). Unlike the covalent phosphorothioate modification in the DNA backbone (21–24), a mass spectrometry analysis further determined that DNA isolated from GFAJ-1 cells grown with limiting phosphate and abundant arsenate contains trace amounts of free arsenate but no detected arsenate-conjugated mono- or dinucleotides (25). These data collectively provide evidence that GFAJ-1 is a highly arsenate-tolerant, yet still phosphate-dependent, bacterial strain.

Although it has not been proved that arsenic can be incorporated into biomolecules in strain GFAJ-1, the ability to thrive in 200 mM As(V) makes GFAJ-1 one of the most arsenic-resistant microorganisms described so far. Thus, understanding the molecular details of this bacterium’s arsenic detoxification process is of great interest. In this study, we determined that two unusual arsenic detoxification operons, *arsH1*-*acr3*-*2*-*arsH2* and *mfs1*-*mfs2*-*gapdh*, cooperatively enable the high arsenate resistance of GFAJ-1. The gene products of *mfs2* and *gapdh*, which resemble ArsJ and glyceraldehyde-3-phosphate dehydrogenase (GAPDH) in Pseudomonas aeruginosa, respectively, catalyze the transformation of inorganic As(V) to organoarsenical 1-arseno-3-phosphoglycerate (1As3PGA), which is then transported out of cells (26). Although th*e arsH1*-*acr3*-*2*-*arsH2* operon is merely composed of three typical *ars* genes, it is sufficient to confer both As(III) and As(V) resistance on Escherichia coli AW3110 without the involvement of arsenate reductase, suggesting a novel As(V) detoxification mechanism. The simultaneous occurrence of these two distinctive arsenic resistance pathways enables GFAJ-1 to directly detoxify arsenate, which explains its arsenate hyperresistance and adaptation to Mono Lake, a particularly arsenic-rich environment.

## RESULTS

### Genome characterization and mining of arsenic-related genes in *Halomonas* sp. strain GFAJ-1.

**FIG 1:**
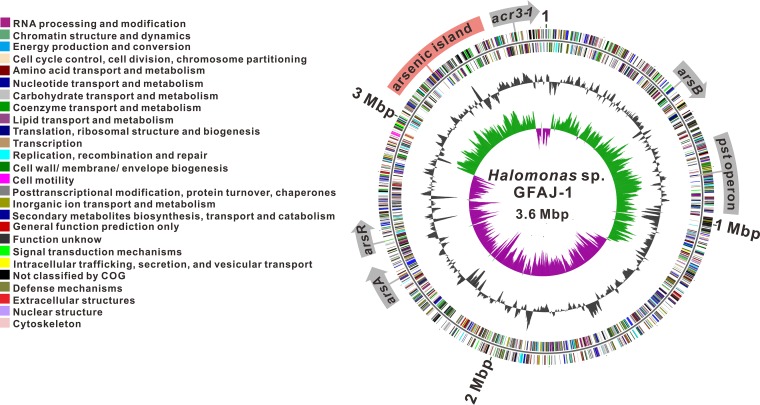
Schematic representation of arsenic-related genes across the complete *Halomonas* sp. strain GFAJ-1 genome. The arsenic island and sporadically distributed putative arsenic resistance determinants are displayed on the GFAJ-1 genome. The circles display the following: circles 1 and 2 (from the outside inward) (forward and reverse strands, respectively), predicted protein-coding sequences, colored according to COG (clusters of orthologous groups) functional categories; circles 3 and 4, GC content and GC skew, respectively.

To explore the genes determining arsenic resistance, we first set out to define the complete genome sequence of the GFAJ-1 strain using the HiSeq 2000 sequencing platform. The sequence reads were *de novo* assembled with the SOAP (short oligonucleotide analysis package) assembler (http://soap.genomics.org.cn/), which generated a single circular chromosome of 3,650,492 bases, 3,385 open reading frames (ORFs), 69 tRNAs, and 36 rRNAs (Fig. 1). The complete genome was annotated with the NCBI Prokaryotic Genome Annotation Pipeline, submitted to NCBI, and assigned GenBank accession number CP016490.

**FIG 2:**
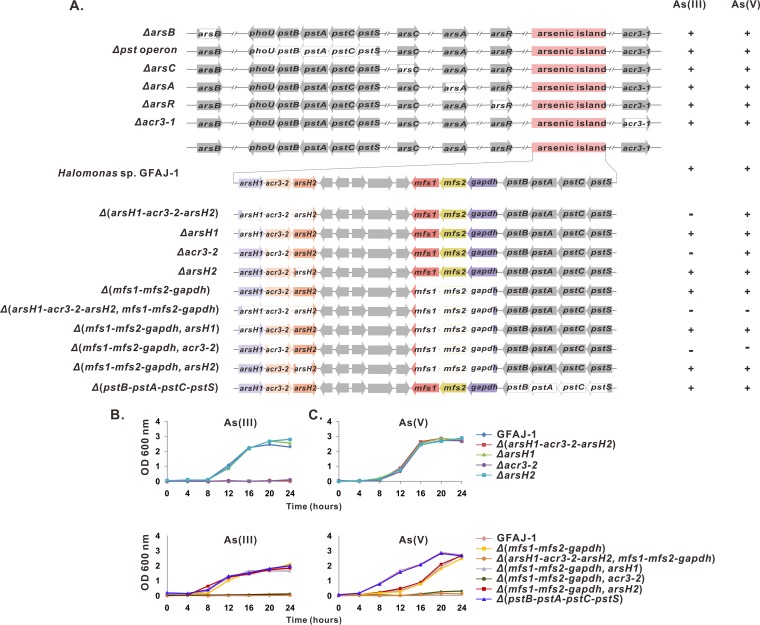
Characterization of arsenic resistance in deletion mutants. (A) Diagram of the arsenic-related genes in strain GFAJ-1 and their effects on resistance to As(III) and As(V). (B and C) Growth profiles of a series of mutants in amended AML60 medium supplemented with either 5 mM As(III) or 200 mM As(V). OD 600 nm, optical density at 600 nm.

Compared to the previous draft genome of strain GFAJ-1 (AHBC00000000), which comprises 103 contigs (27), the current version of the complete GFAJ-1 genome enabled the mining of all potential arsenic-related genes. A region highly resembling an “arsenic island” such as those found in other *Halomonas* species was immediately identified (Fig. 1) (27). This arsenic island comprises a group of typical arsenic resistance determinants, a three-gene *ars* operon (*arsH1*, *acr3*-*2*, and *arsH2*), a phosphate-specific transport (*pst*) operon (*pstB*, *pstA*, *pstC*, and *pstS*), transporters of the major facilitator superfamily (MFS) (*mfs1* and *mfs2*), and a putative glyceraldehyde-3-phosphate dehydrogenase (*gapdh*) (Fig. 2). ACR3 is a member of the bile/arsenite/riboflavin transporter (BART) superfamily, which has a function similar to that of ArsB in extruding As(III) from the cytosol. The gene products of *arsH1* and *arsH2* were both predicted to be NADPH-dependent flavin mononucleotide (FMN) reductases but shared little sequence identity with each other. The structure of ArsH from Sinorhizobium meliloti has been resolved, and ArsH has been characterized as a reductase with diverse substrates; this enzyme has been reported to participate in the reduction of azo dyes and O_2_ to H_2_O_2_, the reduction of chromate Cr(VI) to Cr(III) and Fe(III) to Fe(II), and the reduction of quinones, though not the reduction of inorganic As(V) (28–31). A recent study showed that ArsH from Pseudomonas putida confers resistance to the trivalent forms of organoarsenicals by oxidizing them to the relatively less toxic pentavalent forms, suggestive of the role of a trivalent organoarsenical oxidase (32). In addition to the *pst* genes in the arsenic island, GFAJ-1 possesses an additional *pst* operon mediating arsenate uptake. However, Elias et al. revealed that the two *pst* systems of strain GFAJ-1 still displayed an exquisite specificity for phosphate, ruling out the possibility that they had diverged to import arsenate under phosphate-limiting and arsenate-rich conditions (33). This result agreed well with our observation that the deletion of either *pst* operon in strain GFAJ-1 showed no effect on arsenic resistance. Additional genes, such as annotated *arsA*, *arsB*, *arsR*, and *acr3*-*1*, were also found scattered throughout the GFAJ-1 chromosome (Fig. 1).

### Determinants involved in the As(III) resistance of strain GFAJ-1.

**FIG 3:**
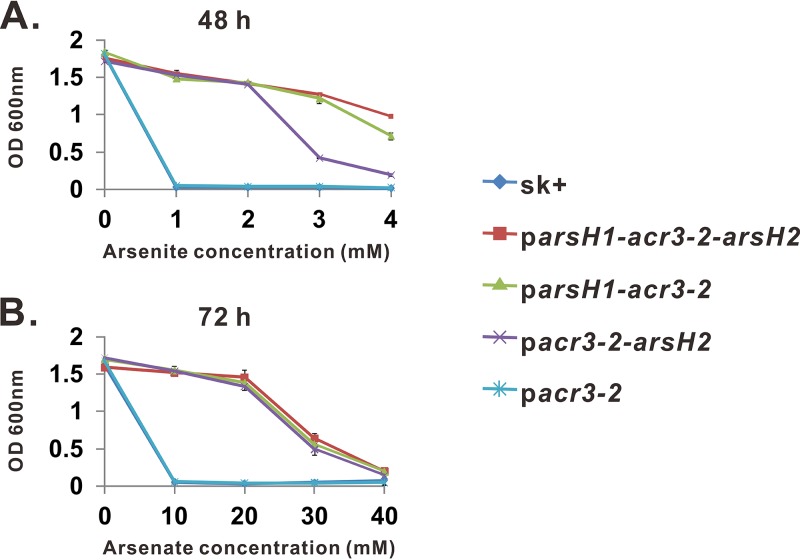
Growth of *E*. *coli* AW3110 bearing different versions of the *ars* operon in the presence of As(III) and As(V). Arsenite and arsenate resistance assays were conducted in LB medium and low-phosphate medium, respectively. The optical density (OD) at 600 nm was determined at the indicated culture times. Error bars represent the standard deviations (SD) from three independent assays.

The presence of two typical As(III) efflux permeases, ArsAB and ACR3, in the GFAJ-1 genome implies the existence of As(III) detoxification pathways, which have been recognized as essential for both As(III) and As(V) tolerance in many bacteria. To confirm the determinants of As(III) resistance, we first performed in-frame deletions of the genes related to As(III) efflux pumps, generating the *ΔarsA*, *ΔarsB*, *ΔarsR*, *Δacr3*-*1*, and *Δacr3*-*2* mutants. No differences were observed between the wild-type GFAJ-1 strain and the *ΔarsA*, *ΔarsB*, *ΔarsR*, or *Δacr3*-*1* mutant in their susceptibilities to 5 mM As(III), indicating that these scattered annotated *ars* genes are not involved in arsenic resistance (Fig. 2). The deletion of *acr3*-*2*, however, completely abolished As(III) resistance, suggesting its key function in the As(III) detoxification of GFAJ-1 (Fig. 2). *arsH1*, *acr3*-*2*, and *arsH2* are cotranscribed as a single operon, and their heterologous expression conferred resistance to As(III) on the arsenic-sensitive E. coli strain AW3110, indicating their connected functions in the arsenic detoxification process (see Fig. S1A in the supplemental material). To evaluate the contribution of each *arsH* gene to As(III) tolerance, different combinations of the *ars* genes, *arsH1*-*acr3*-*2*, *acr3*-*2*, and *acr3*-*2*-*arsH2*, were introduced separately into E. coli AW3110. As shown in Fig. 3A, the combination of *acr3*-*2* and either *arsH* conferred As(III) resistance on AW3110 cells, but *arsH1*-*acr3*-*2* cells showed a much higher level of resistance than *acr3*-*2*-*arsH2* cells did. These results clearly demonstrate that the *arsH1*-*acr3*-*2*-*arsH2* cluster is the functional determinant of As(III) tolerance in strain GFAJ-1. Notably, E. coli AW3110 bearing *acr3*-*2* alone was still sensitive to As(III), suggesting that Acr3-2 requires ArsH to synergistically detoxify As(III).

10.1128/mBio.00515-18.2 FIG S1  Reverse transcription-PCR (RT-PCR) analysis of the cotranscription of the *arsH1*-*acr3*-*2*-*arsH2* operon and the *mfs1*-*mfs2*-*gapdh* operon. Primers are schematically located above or below the genes. PCR products were obtained using reverse-transcribed cDNA (lanes 1 and 5), genomic DNA (lanes 2 and 6), and nontranscribed RNA (lanes 3 and 7) of strain GFAJ-1 as the templates. Primers are listed in Table S2 in the supplemental material. Download FIG S1, TIF file, 2.6 MB. Copyright © 2018 Wu et al. 2018 Wu et al. This content is distributed under the terms of the Creative Commons Attribution 4.0 International license.

### Direct As(V) detoxification mediated by the *mfs1*-*mfs2*-*gapdh* cluster in strain GFAJ-1.

Before the primordial atmosphere became oxidizing, As(III) is thought to have been the predominant arsenic species, driving early microorganisms to evolve detoxification mechanisms to cope with As(III). Pentavalent As(V) would have become the major arsenic form when oxygen appeared in the atmosphere. To adapt to this toxic pressure, arsenate reductase arose to transform As(V) to As(III) for subsequent detoxification via the existing As(III) extrusion pathways (34). This evolutionary history explains why organisms convert the less toxic As(V) to the more toxic As(III) as part of detoxification. Surprisingly, we observed that the *Δ*(*arsH1*-*acr3*-*2*-*arsH2*) mutant, lacking the essential As(III) extrusion pathway, was sensitive to As(III) but still highly resistant to As(V), implying alternative arsenate resistance mechanisms (Fig. 2).

Recently, a case of As(V) efflux activity was observed in which ArsJ and GAPDH from Pseudomonas aeruginosa DK2 synergistically reduce cellular inorganic As(V) accumulation via a two-step reaction. In the first step, arsenate substitutes for phosphate in glycolysis, generating pentavalent organoarsenical 1-arseno-3-phosphoglycerate (1As3PGA) via GAPDH. In the following step, ArsJ, a major facilitator superfamily (MFS) protein, functions as an organoarsenical efflux permease to extrude 1As3PGA from cells, where the unstable 1As3PGA rapidly hydrolyzes to As(V) and 3-phosphoglycerate, making ArsJ and GAPDH an equivalent As(V) efflux system (26).

**FIG 4:**
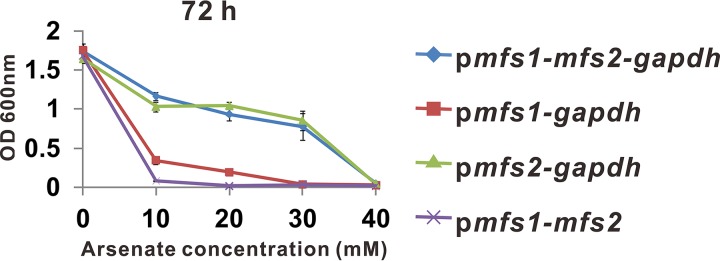
Growth of E. coli AW3110 containing different versions of the *mfs1*-*mfs2*-*gapdh* operon in the presence of As(V). Arsenate exposure was conducted in low-phosphate medium. The OD_600_ was determined at the indicated culture times. Error bars represent the SD of three independent assays.

Given the observation that mutation of the As(III) efflux pathway had a negligible impact on the As(V) resistance of strain GFAJ-1, as well as the presence of the *mfs1*-*mfs2*-*gapdh* operon in the “arsenic island,” a similar direct As(V) efflux mechanism may be feasible in GFAJ-1. A sequence comparison revealed that the gene products of *mfs2* and *gapdh* are highly homologous to ArsJ and GAPDH in P. aeruginosa DK2, sharing 63% and 72% sequence identity, respectively. *mfs1* is also a putative MFS protein-encoding gene, and its gene product was predicted to possess 10 transmembrane-spanning segments, similar to MFS2, using the TMHMM Server v.2.0 (http://www.cbs.dtu.dk/services/TMHMM/). MFS1 showed no significant sequence similarity to ArsJ or MFS2, but its contextual affiliation with *mfs2* and *gapdh* suggested a connected function in arsenic detoxification (Fig. S1B). To investigate As(V) extrusion in strain GFAJ-1, we constructed a *Δ*(*mfs1*-*mfs2*-*gapdh*) mutant and assessed its susceptibility to arsenate. The *Δ*(*mfs1*-*mfs2*-*gapdh*) mutant remained resistant to As(V) but showed an apparent lag of approximately 8 h before undergoing visible growth in liquid broth supplemented with 200 mM As(V), in contrast to the behavior of wild-type GFAJ-1 (Fig. 2). As expected, the deletion of *mfs1*-*mfs2*-*gapdh* had no effect on As(III) resistance. Despite the cotranscription of *mfs1*, *mfs2*, and *gapdh*, the combination of *mfs1* and *gapdh* conferred negligible arsenate resistance on E. coli AW3110 cells. In sharp contrast, E. coli AW3110 harboring *mfs2*-*gapdh* and *mfs1*-*mfs2*-*gapdh* showed equivalent levels of resistance to As(V) (Fig. 4). These results confirmed that the As(V) efflux detoxification mediated by the *mfs1*-*mfs2*-*gapdh* operon is functional and partially contributes to the As(V) hyperresistance of strain GFAJ-1.

### A novel As(V) resistance strategy.

Without an As(V) extrusion system, the *Δ*(*mfs1*-*mfs2*-*gapdh*) mutant is still resistant to As(V), implying multiple arsenic detoxification pathways. To address this issue, we generated a double mutant carrying the *arsH1*-*acr3*-*2*-*arsH2* mutation in the *Δmfs1*-*mfs2*-*gapdh* background. As shown in Fig. 2, this *Δ*(*arsH1*-*acr3*-*2*-*arsH2*, *mfs1*-*mfs2*-*gapdh*) mutant was severely growth sensitive to As(V), suggesting the vital role of the *arsH1*-*acr3*-*2*-*arsH2* cluster in As(V) detoxification. We then set out to locate a possible arsenate reductase, which is thought to cooperate with ArsH1, ACR3-2, and ArsH2 to catalyze As(V) reduction and As(III) extrusion. Upon exploring the GFAJ-1 genome for genes encoding arsenate reductases, we detected none in the vicinity of the arsenic resistance island. However, one gene annotated as *arsC* in a region unrelated to arsenic transformation or handling was identified (Fig. 2A). To assess its possible role as an arsenate reductase, we constructed the *Δ*(*mfs1*-*mfs2*-*gapdh*, *arsC*) double mutant to simultaneously delete the As(V) efflux and putative As(V) reduction pathways. Surprisingly, no difference was observed in its As(V) resistance phenotype, implying that this annotated *arsC* does not encode a functional arsenate reductase.

Considering the necessity and unusual composition of the *ars* operon in As(V) detoxification, we tested the As(V) tolerance of E. coli AW3110 bearing *arsH1*-*acr3*-*2*, *acr3*-*2*, and *acr3*-*2*-*arsH2*. Surprisingly, even in the absence of arsenate reductase, the *ars* operon is sufficient to detoxify As(V) (Fig. 3B). As in As(III) resistance, single ACR3-2 was unable to confer As(V) resistance on E. coli AW3110 unless combined with ArsH. This result agreed well with the observation that *Δ*(*mfs1*-*mfs2*-*gapdh*, *arsH1*) and *Δ*(*mfs1*-*mfs2*-*gapdh*, *arsH2*) mutants showed growth profiles similar to that of the *Δ*(*mfs1*-*mfs2*-*gapdh*) mutant in the presence of As(V) (Fig. 2). Therefore, ArsH1, ACR3-2, and ArsH2 represent a novel As(V) detoxification strategy that does not require the involvement of arsenate reduction. The As(V) hyperresistance of strain GFAJ-1 is attributable to the simultaneous occurrence of two unusual As(V) detoxification mechanisms involving both *arsH1*-*acr3*-*2*-*arsH2* and *mfs1*-*mfs2*-*gapdh*.

## DISCUSSION

Following the original announcement of possible replacement of phosphorus by arsenic in strain GFAJ-1, scientists sought to confirm this finding by multiple approaches, and eventually, GFAJ-1 was found to contain no arsenic in its DNA following the reported conditions. On the other hand, the ability to thrive in 200 mM As(V) makes GFAJ-1 one of the most arsenic-resistant microorganisms described thus far, prompting us to explore the mechanisms of its hyperresistance. Upon deletion of *acr3*-*2*, the arsenite-specific transporter, the *Δacr3*-*2* mutant is susceptible to As(III), suggesting the occurrence of an As(III) extrusion mechanism and possible arsenate reduction in As(V) resistance. Genome sequencing and mining revealed only one gene annotated as the arsenate reductase ArsC in a region unrelated to arsenic metabolism; mutagenesis showed that this gene is not a functional arsenate reductase and has no influence on As(V) resistance in strain GFAJ-1. This phenomenon raises two possibilities. (i) A functional arsenate reductase is present in strain GFAJ-1 but has not yet been located owing to a low sequence similarity to known ArsCs and other types of arsenate reductase. (ii) Arsenate reduction activity does not occur in GFAJ-1. Our study supports the latter possibility, because the *arsH1*-*acr3*-*2*-*arsH2* gene products are sufficient to confer both As(III) and As(V) resistance on E. coli AW3110 without the involvement of any arsenate reductase.

ACR3-2 is an arsenite-specific efflux carrier and is essential to detoxify As(III) in strain GFAJ-1. Compared to other well-defined *ars* determinants, *arsH* is a relatively newly discovered *ars* component, and it exhibits a variety of effects on arsenical resistance in different microorganisms. For instance, ArsH provides a slight increase in both As(III) and As(V) resistance in Yersinia enterocolitica, *arsH* inactivation increased sensitivity to low levels of As(III) and As(V) in Sinorhizobium meliloti, and the mutation or expression of *arsH* in *Synechocystis* sp. strain PCC 6803 and Thiobacillus ferrooxidans does not appear to influence arsenic resistance (35–38). In terms of biological function, ArsH exhibits the ability to reduce azo dyes, chromate, ferric ion, and quinones but not arsenate (28–31). Chen et al. (32) recently reported that ArsH from Pseudomonas putida acts as an oxidase to confer high-level resistance to trivalent organoarsenicals, e.g., monosodium methylarsenate, roxarsone (3-nitro-4-hydroxyphenylarsonic acid), and phenylarsenite, by oxidizing them to the relatively less toxic pentavalent species. However, no oxidation activity by ArsH was observed when inorganic As(III) was used as a substrate (32). The individual *acr3*-*2* gene from strain GFAJ-1 is insufficient to confer As(III) or As(V) resistance to E. coli unless it is combined with an *arsH* gene, suggesting the essential role of *arsH* in arsenic detoxification. Both the combinations *arsH1*-*acr3*-*2* and *arsH2*-*acr3*-*2* increase arsenic resistance in E. coli AW3110, but the former confers the ability to tolerate higher levels of As(III) than the latter does, indicating the contributions of ArsH1 and ArsH2 to high-level and low-level resistance, respectively. It is unlikely that the *arsH* gene products in strain GFAJ-1 perform As(V) reduction, because the deletion of *arsH1* or *arsH2* in the *Δ*(*mfs1*-*mfs2*-*gapdh*) mutant did not result in an increased arsenate sensitivity, whereas the *Δ*(*acr3*-*2*, *mfs1*-*mfs2*-*gapdh*) mutant was completely susceptible to arsenate. As to the absence of arsenate reductase, one plausible explanation is that ArsH1-ACR3-2-ArsH2 creates a new detoxification pathway to bypass the As(V) reduction step. Currently, whether ArsH1-ACR3-2-ArsH2 extrudes As(V) out of cells directly or catalyzes the conversion of As(V) to an intermediate prior to the efflux remains unclear.

Although evolution has determined the conventional *ars* detoxification mechanism, in which As(V) is transformed to As(III) and As(III) is subsequently extruded, a direct arsenate efflux system would benefit cells, because it would not require the involvement of reductase, and As(V) would not be converted to the more toxic As(III). Chen et al. recently identified an arsenate efflux pathway in P. aeruginosa: GAPDH converts inorganic arsenate to the organoarsenical 1As3PGA, which is subsequently extruded from cells by the pentavalent organoarsenical efflux permease ArsJ, an MFS superfamily protein (26). Such a direct As(V) efflux pathway also occurs in strain GFAJ-1, based on the observations that MFS2 and GAPDH in strain GFAJ-1 show high sequence similarities to P. aeruginosa ArsJ and GAPDH, respectively, and that the disruption of *mfs2* and *gapdh* in the *Δ*(*arsH1*-*acr3*-*2*-*arsH2*) background either abolished or remarkably impaired arsenate resistance. Further investigation is needed to characterize the potential involvement of arsenic in biological processes such as energy generation or electron transfer in strain GFAJ-1 under phosphate-limited conditions. Collectively, our data revealed that the arsenate hyperresistance of strain GFAJ-1 is attributable to the cooccurrence of two arsenate detoxification pathways and that these pathways directly detoxify arsenate instead of performing the typical conversion of As(V) to As(III) and extrusion of As(III).

## MATERIALS AND METHODS

### Bacterial strains, plasmids, and growth conditions.

The strains and plasmids mentioned in this study are listed in Table S1 in the supplemental material. E. coli DH10B and E. coli AW3110 were used as hosts for plasmid construction and the expression of arsenic genes, respectively. The *Halomonas* strains were grown at 28°C in AML60 liquid broth by the method of Wolfe-Simon et al. (19), supplemented with 0.2 g/liter yeast extract but no added vitamins, whereas E. coli DH10B and derivatives were cultured in Luria-Bertani (LB) broth or on agar plates at 37°C. E. coli AW3110 strains expressing arsenic-related genes displayed better growth in LB or low-phosphate medium (36) at 28°C compared to 37°C.

10.1128/mBio.00515-18.1 TEXT S1  Supplemental information Materials and Methods and references. Download TEXT S1, DOCX file, 0.02 MB. Copyright © 2018 Wu et al. 2018 Wu et al. This content is distributed under the terms of the Creative Commons Attribution 4.0 International license.

10.1128/mBio.00515-18.3 TABLE S1  Strains used in this study. Download TABLE S1, DOCX file, 0.02 MB. Copyright © 2018 Wu et al. 2018 Wu et al. This content is distributed under the terms of the Creative Commons Attribution 4.0 International license.

10.1128/mBio.00515-18.4 TABLE S2  Primers used in this study. The enzymatic sites are shown in red. Download TABLE S2, DOCX file, 0.02 MB. Copyright © 2018 Wu et al. 2018 Wu et al. This content is distributed under the terms of the Creative Commons Attribution 4.0 International license.
