# Supplementary figures and images for: Signature Arsenic Detoxification Pathways in Halomonas sp. Strain GFAJ-1
Source: mBio. 2018 May 1;9(3):e00515-18. doi: 10.1128/mBio.00515-18 (PMC5930303; doi:10.1128/mBio.00515-18)

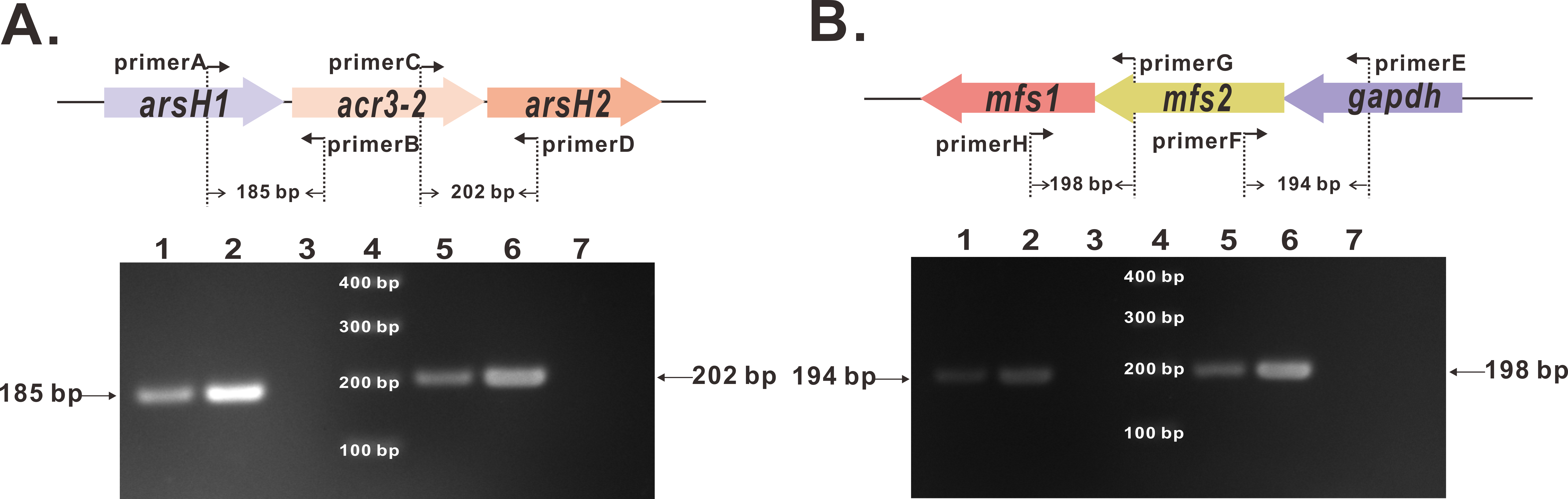

Supplement: FIG S1 [file mbo002183827sf1.tif]
