# Supplementary material for: Signature Arsenic Detoxification Pathways in Halomonas sp. Strain GFAJ-1
Source: mBio. 2018 May 1;9(3):e00515-18. doi: 10.1128/mBio.00515-18 (PMC5930303; doi:10.1128/mBio.00515-18)
Supplement: TEXT S1 [file mbo002183827s1.docx]

**Supplementary Information**

**Signature arsenic detoxification pathways**

**in *Halomonas* sp. GFAJ-1**

**SI MATERIALS AND METHODS**

**Whole genome sequencing of GFAJ-1**

The genome of GFAJ-1 was sequenced using the Illumina HiSeq 2000 sequencing platform at the Beijing Genome Institute (Shenzhen, China). Three libraries harboring 500 bp, 2000 bp and 6000 bp inserts were constructed, generating 651, 535 and 851 Mb of raw data, respectively. The resulting sequence reads were ﬁltered and assembled using the SOAPdenovo 1.05 software package (1). Genes were predicted from the assembled GFAJ-1 genome using Glimmer 3.02. Gene functions were annotated by BLASTp analysis with the Gene Ontology (GO), Kyoto Encyclopedia of Genes and Genomes (KEGG), Swiss-Prot, TrEMBL and Clusters of Orthologous Groups (COG) databases.

**Construction of GFAJ-1 mutants**

To identify the determinants involved in arsenic resistance, a series of in-frame deletion *Halomonas* mutants were constructed using a two-step homologous recombination procedure. First, the DNA fragments upstream and downstream of a target gene were PCR amplified using two pairs of primers (listed in Table S2). Second, the recombinant fragment was generated using a mixture of the upstream and downstream PCR products (with an overlap of approximately 40 bp) as templates and then ligated to SpeI- and SacI-digested pJC4, a suicide vector. In this way, pJC4 derivatives were generated and transformed into DAP (diaminopimelic acid) auxotroph *E. coli* WM3064, which was then conjugated to *Halomonas*. The transconjugants bearing suicide plasmids were used for double-crossovers on an LB agar plate containing 10-15% sucrose at 28°C. The in-frame deletions of the target genes were confirmed by PCR and sequencing.

**Construction of plasmids expressing arsenic-related genes**

To obtain different versions of the *ars* operon, DNA fragments containing *arsH1-acr3-2-arsH2*, *arsH1-acr3-2*, and *acr3-2-arsH2* were PCR amplified from the genomic DNA of wild-type GFAJ-1, *ΔarsH2* and *ΔarsH1*, respectively, using HEars-f and HEars-r as primers. The fragment harboring *acr3-2* alone was PCR amplified using the genomic DNA of *ΔarsH1* as the template and HEacr3-2-f and HEacr3-2-r as primers (Table S2). The resulting PCR products were subsequently inserted into the pBluescript II SK+ plasmid to generate the plasmids pWHU764, pWHU3356, pWHU3357 and pWHU3358, expressing *arsH1-acr3-2-arsH2*, *arsH1-acr3-2*, *acr3-2-arsH2,* and *acr3-2*, respectively. Similarly, to obtain different versions of the *mfs1-mfs2-gapdh* operon, fragments containing *mfs1-mfs2-gapdh, mfs1-gapdh*, *mfs2-gapdh*, *mfs1-mfs2* were PCR amplified from the genomic DNA of GFAJ-1, *Δmfs2*, *Δmfs1* and *Δgapdh*, respectively, using HEmfs-f and HEmfs-r as primers. The PCR products were cloned into pEASY-Blunt Zero Cloning Vector (Transgen Biotech, Beijing), yielding pWHU3203, pWHU3204 pWHU3205 and pWHU3206, respectively. All constructs of arsenic-related genes were under the control of the native promoters.

**Arsenic resistance assays**

For the arsenite resistance assays in liquid medium, *E. coli* AW3110 bearing the indicated plasmids was grown in LB medium, while for arsenate resistance, cells were cultured in low-phosphate medium. Overnight cultures were diluted 100-fold into medium containing different concentrations of either arsenite or arsenate and were incubated at 28°C with shaking. Cell growth was estimated from the absorbance at 600 nm.

**RNA isolation and RT-PCR analysis**

Total RNA was isolated from mid-exponential-phase GFAJ-1 using the RNeasy Mini Kit (QIAGEN) and treated with RNase-free DNase I (Thermo) to remove residual DNA. Reverse transcription (RT)-PCR was conducted using the RevertAid First Strand cDNA Synthesis kit (Thermo) according to the manufacturer’s instructions.

**Figure Legend**

**Figure S1. RT-PCR analysis of the co-transcription of *arsH1-acr3-2-arsH2* operon and *mfs1-mfs2-gapdh* operon, respectively.** Primers are schematically located above or below the genes. PCR products were obtained using reverse-transcribed cDNA (lane 1, 5), genomic DNA (lane 2, 6) and non-transcribed RNA (lane 3, 7) of GFAJ-1 as templates. Primers are listed in Table S2.

**SI REFERENCES**

1. Li R, Li Y, Kristiansen K, Wang J. 2008. SOAP: short oligonucleotide alignment program. Bioinformatics 24:713-4.

2. Dehio C, Meyer M. 1997. Maintenance of broad-host-range incompatibility group P and group Q plasmids and transposition of Tn5 in Bartonella henselae following conjugal plasmid transfer from *Escherichia coli*. J Bacteriol 179:538-40.

3. Carlin A, Shi W, Dey S, Rosen BP. 1995. The ars operon of *Escherichia coli* confers arsenical and antimonial resistance. J Bacteriol 177:981-6.

4. Wolfe-Simon F, Switzer Blum J, Kulp TR, Gordon GW, Hoeft SE, Pett-Ridge J, Stolz JF, Webb SM, Weber PK, Davies PC, Anbar AD, Oremland RS. 2011. A bacterium that can grow by using arsenic instead of phosphorus. Science 332:1163-6.

5. Alting-Mees MA, Short JM. 1989. pBluescript II: gene mapping vectors. Nucleic Acids Res 17:9494.
