# Supplementary material for: Signature Arsenic Detoxification Pathways in Halomonas sp. Strain GFAJ-1
Source: mBio. 2018 May 1;9(3):e00515-18. doi: 10.1128/mBio.00515-18 (PMC5930303; doi:10.1128/mBio.00515-18)
Supplement: TABLE S1 [file mbo002183827st1.docx]

**Table S1. Strains used in this study**

| **Strains/plasmids** | **Characteristics** | **Source/Reference** | | |
| --- | --- | --- | --- | --- |
| **Strains** |  | | |  |
| *E. coli* WM3064 | donor strain λpir, RP4 (tra) in chromosome, DAP^-^ | | | (2) |
| *E. coli* DH10B | *E. coli* host for plasmid construction and replication | | | Invitrogen |
| *E. coli* AW3110 | K-12 F- IN(rrnD-rrnE)Δ*ars*::cam | | | (3) |
| *Halomonas* sp. GFAJ-1 | arsenic resistant | | | (ATCC BAA-2256) (4) |
| *Δ*(*arsH1-acr3-2-arsH2*) | GFAJ-1 derivative with *ars* cluster deleted | | | This work |
| *ΔarsH1* | GFAJ-1 derivative, *arsH1* in-frame deletion mutant | | | This work |
| *Δacr3-2* | GFAJ-1 derivative, *acr3-2* in-frame deletion mutant | | | This work |
| *ΔarsH2* | GFAJ-1 derivative, *arsH2* in-frame deletion mutant | | | This work |
| *ΔarsA* | GFAJ-1 derivative, *arsA* in-frame deletion mutant | | | This work |
| *ΔarsB* | GFAJ-1 derivative, *arsB* in-frame deletion mutant | | | This work |
| *ΔarsC* | GFAJ-1derivative, *arsC* in-frame deletion mutant | | | This work |
| *ΔarsR* | GFAJ-1derivative, *arsR* in-frame deletion mutant | | | This work |
| *Δacr3-1* | GFAJ-1derivative, *acr3-1* in-frame deletion mutant | | | This work |
| *Δ*(*mfs1-mfs2-gapdh*) | GFAJ-1 derivative, *mfs1-mfs2-gapdh* in-frame deletion mutant | | | This work |
| *Δ*(*arsH1-acr3-2-arsH2*, *mfs1-mfs2-gapdh*) | GFAJ-1 derivative with in-frame deletion of *arsH1-acr3-2-arsH2* and *mfs1-mfs2-gapdh* clusters | | | This work |
| *Δ*(*mfs1-mfs2-gapdh, arsH1*) | *Δ*(*mfs1-mfs2-gapdh*) mutant derivative, *arsH1* in-frame deletion mutant | | | This work |
| *Δ*(*mfs1-mfs2-gapdh, acr3-2*) | *Δ*(*mfs1-mfs2-gapdh*) mutant derivative, *acr3-2* in-frame deletion mutant | | | This work |
| *Δ*(*mfs1-mfs2-gapdh, arsH2*) | *Δ*(*mfs1-mfs2-gapdh*) mutant derivative, *arsH2* in-frame deletion mutant | | | This work |
| *Δ*(*pstB-pstA-pstC-pstS*) | GFAJ-1 derivative, *pstB-pstA-pstC-pstS* in-frame deletion mutant | | | This work |
| **Plasmids** |  | | |  |
| pBluescript II SK+ | Amp^r^, lacZ, cloning vector | | | (5) |
| pEASY-Blunt Zero | Amp^r^, lacZ, cloning vector | | Transgen Biotech | |
| pWHU764 | SK+ derivative carrying *arsH1*-*acr3-2*-*arsH2* fragment | | | This work |
| pWHU3356 | SK+ derivative carrying *arsH1*-*acr3-2* fragment | | | This work |
| pWHU3357 | SK+ derivative carrying *acr3-2*-*arsH2* fragment | | | This work |
| pWHU3358 | SK+ derivative carrying *acr3-2* fragment | | | This work |
| pWHU3203 | pEASY-Blunt Zero derivative carrying *mfs1-mfs2-gapdh* fragment | | | This work |
| pWHU3204 | pEASY-Blunt Zero derivative carrying *mfs1-gapdh* fragment | | | This work |
| pWHU3205 | pEASY-Blunt Zero derivative carrying *mfs2-gapdh* fragment | | | This work |
| pWHU3206 | pEASY-Blunt Zero derivative carrying *mfs1-mfs2* fragment | | | This work |
