# Supplementary material for: Signature Arsenic Detoxification Pathways in Halomonas sp. Strain GFAJ-1
Source: mBio. 2018 May 1;9(3):e00515-18. doi: 10.1128/mBio.00515-18 (PMC5930303; doi:10.1128/mBio.00515-18)
Supplement: TABLE S2 [file mbo002183827st2.docx]

**Table S2. Primers used in this study**

| **Primers** | **Sequences (5’-3’)** |
| --- | --- |
| **Construction of *Δ*(*arsH1-acr3-2-arsH2*)** | |
| ars cluster-1 | GAGCTCCCAGCCGCTCAACGATTT |
| ars cluster-2 | GGATTTATAGTTGCCAATAGGCTGTGGCTAGGAACACTAAAGGA |
| ars cluster-3 | TCCTTTAGTGTTCCTAGCCACAGCCTATTGGCAACTATAAATCC |
| ars cluster-4 | ACTAGTCACCTTTGAAGCGGTACTTT |
| **Construction of *ΔarsH1*** | |
| arsH1-1 | GAGCTCTAACCTCGCCCACCTCAC |
| arsH1-2 | ATCACTGCGCTCACGAACCAGCGTTTCATGGTTAGGC |
| arsH1-3 | GCCTAACCATGAAACGCTGGTTCGTGAGCGCAGTGAT |
| arsH1-4 | ACTAGTGCAAAGGCAAACAGCATAA |
| **Construction of *Δacr3-2*** |  |
| acr3-2-1 | GAGCTCGAACGCTCTTTCAGCCGATTA |
| acr3-2-2 | GACGAGGACACCAACGACTGCAATTCCAGCGACAAT |
| acr3-2-3 | ATTGTCGCTGGAATTGCAGTCGTTGGTGTCCTCGTC |
| acr3-2-4 | ACTAGTTCTTGTTCGGCTTTGTGC |
| **Construction of *ΔarsH2*** |  |
| arsH2-1 | GAGCTCAGCGATGGTATTTGTATGGA |
| arsH2-2 | GGTCTTGTTCGGCTTTGTGGCGTGTTGAGCCAAGAAA |
| arsH2-3 | TTTCTTGGCTCAACACGCCACAAAGCCGAACAAGACC |
| arsH2-4 | ACTAGTGTGAAGATGGGCTTATGGA |
| **Construction of *Δ*(*mfs1-mfs2-gapdh*)** | |
| mfs cluster-1 | GAGCTCACTCACAATATGCAGCAAG |
| mfs cluster-2 | GGGTGCGAGTGCTTGAGACACATCGCAGCCAGACCA |
| mfs cluster-3 | TGGTCTGGCTGCGATGTGTCTCAAGCACTCGCACCC |
| mfs cluster-4 | ACTAGTACCAGCACTGTCGCTACCC |
| **Construction of *Δmfs1*** | |
| mfs1-1 | GAGCTCATGGCGGTGATGCTTGGC |
| mfs1-2 | GGGTGCGAGTGCTTGAGAAAGAATGGCGGGCAAATA |
| mfs1-3 | TATTTGCCCGCCATTCTTTCTCAAGCACTCGCACCC |
| mfs1-4 | ACTAGTGTCCTGGCGGTAGGTGAT |
| **Construction of *Δmfs2*** |  |
| mfs2-1 | GAGCTCGACCATCTTTACGACCCTG |
| mfs2-2 | CAACCAGCCTGACAGCAACGAGTAACCTAGCTGGTGAAA |
| mfs2-3 | TTTCACCAGCTAGGTTACTCGTTGCTGTCAGGCTGGTTG |
| mfs2-4 | ACTAGTCTGCCACCGAAAGCGATA |
| **Construction of *Δgapdh*** |  |
| gapdh-1 | GAGCTCGACTACACCGCGTTTATG |
| gapdh-2 | TACCAGTGGGCGATCTTCCACATCGCAGCCAGACCA |
| gapdh-3 | TGGTCTGGCTGCGATGTGGAAGATCGCCCACTGGTA |
| gapdh-4 | ACTAGTCACCACGGAAACCAATCA |
| **Expression of *arsH1-acr3-2-arsH2*, *arsH1-acr3-2*, *acr3-2-arsH2*** | |
| HEars-f | GAATTCCCTTTAGTGTTCCTAGCCACAT |
| HEars-r | GGATCCGTGCATCGACCGTTGCTT |
| **Expression of *acr3-2*** |  |
| HEacr3-2-f | GAATTCTCGCCACGGATATGTCTGCCG |
| HEacr3-2-r | GGATCCCGGCGTAGAAGTGCGTGT |
| **Expression of *mfs1-mfs2-gapdh*, *mfs1-gapdh*, *mfs2-gapdh, mfs1-mfs2*** | |
| HEmfs-f | GCGGCCGCATGACATCGTTGAACAATCG |
| HEmfs-r | GAATTCAAGGCGTCGATGACACAGCC |
| **RT-PCR for *arsH1-acr3-2-arsH2*** | |
| primerA | TCTGCTAACCGACCGCTACTCTG |
| primerB | CGACAATGGCAATCGCTACCC |
| primerC | GCAGCCATTGCGCTGTTTGG |
| primerD | GCGGCGTAGAAGTGCGTGTTG |
|  |  |
| **RT-PCR for *mfs1-mfs2-gapdh*** | |
| primerE | TCGGCACTGAGCAGGTGA |
| primerF | ATTTGCAGCGGCGAGTAA |
| primerG | GGCGTCTCAATGGATGTCG |
| primerH | CAAACCTGGGCGAACGAA |
|  |  |

The enzymatic sites are written in red font.
